# An Aminobenzenethiol-Functionalized Gold Nanocolorimetric Sensor for Formaldehyde Detection

**DOI:** 10.3390/ma17246087

**Published:** 2024-12-13

**Authors:** Jing Xu, Liya Shen, Haining You, Yuanli Liu

**Affiliations:** Guangxi Key Laboratory of Optical and Electronic Materials and Devices, College of Materials Science and Engineering, Guilin University of Technology, Guilin 541004, China; xjclaire@163.com (J.X.); shenliya1999@163.com (L.S.); 13100765957@163.com (H.Y.)

**Keywords:** aminothiophenol, gold nanoparticles, formaldehyde detection, colorimetric sensor

## Abstract

The determination of formaldehyde is of paramount importance, as it is present in numerous locations throughout life. In this study, aminophenol-modified gold nanoparticles (ATP-AuNPs) with different relative positions of hydroxyl and amino groups were synthesized for the detection of formaldehyde. They were characterized by transmission electron microscopy (TEM), ultraviolet–visible (UV-Vis) spectroscopy and Fourier transform infrared (FTIR) spectroscopy tests. The results demonstrated that the position plays a crucial role in the composites, which exhibit good stability when the sulfhydryl group and amino group transition from the para position to the neighboring position. Furthermore, the para position was identified as the optimal configuration for formaldehyde detection. When it was used to detect formaldehyde in ultrapure and Li River water, the limit of detection (LOD) was calculated to be 1.03/1.15 mM, respectively. This work not only provides a novel ATP-AuNP sensor but also highlights its practical situations.

## 1. Introduction

Formaldehyde is readily soluble in water, and a solution of approximately 35.0% to 40.0% formaldehyde (commonly referred to as formalin) possesses potent antiseptic properties, leading to its frequent employment as a preservative and tissue fixative in pharmaceuticals, agriculture and other fields [1,2]. However, formaldehyde solution can enter the human body through food ingestion and other routes of exposure, which can have adverse effects on human health [3]. These effects can range from minor issues such as enlarged laryngeal tissue to more serious conditions such as bronchitis, pneumonia and even asphyxiation [4,5,6]. Consequently, it is imperative that effective measures be implemented to detect formaldehyde in water and to reduce human exposure to this chemical.

At present, more effective strategies are being used for formaldehyde detection. For example, Fu et al. [7] achieved the detection of formaldehyde by constructing a dual-nanosphere electrochemical sensor from gold nanoclusters and polydopamine nanospheres. Teišerskytė et al. [8] constructed a direct electron transfer formaldehyde dehydrogenase biosensor for the determination of formaldehyde in river water. However, they generally face the problems of being time-consuming and having a high cost of instrumentation, so we urgently need to develop a simple and economical method of formaldehyde detection.

Colorimetric assays can enable visual and on-site analysis, providing a simple and instantaneous detection method [9]. Gold nanoparticles respond within a short time after the addition of analytes, and this property has been exploited for colorimetric determination [10]. The performance of the gold nanoparticle colorimetric sensor is contingent upon the variation in the surface plasmon resonance distance. As the gold nanoparticles approach each other, the electromagnetic coupling between them becomes more pronounced, as evidenced by the fact that the gold nanoparticles exhibit different colors in different solution states [11]. Furthermore, the presence of specific molecules or ions in the surface microenvironment may result in interactions with the surfaces of AuNPs [12]. Thereby, modifying the surface charge and energy states of the particles will influence their behavior [13]. For example, Leng et al. [14] presented a Au(I→0) fast reduction-based nanoparticle (AuNP) sensing strategy for the simultaneous and colorimetric detection and differentiation of a wide range of proteins. Yuan et al. [15] constructed a biothiol colorimetric nanosensing array comprising gold nanorods and metal ions (Hg, Pb, Cu, Ag). This innovative approach enabled the researchers to distinguish between five biothiols with the naked eye. Jimenez et al. [16] reported the functionalization of gold nanoparticles with 4-mercaptobenzoic acid in order to modulate the surface plasmon resonance frequency and the absorption of fluorescence energy by AuNPs for the measurement of sugar content in liquids. Thus, functionalized AuNPs can directly respond to the target analytes under investigation with traits such as rapid sensing speed, high reliability, portability and minimal operator training requirements. However, there is a paucity of research on the application of aminobenzenethiol gold nanosurface modification for formaldehyde detection.

Considering the above, in this work, we have rationally designed an efficient and rapid method for the detection of formaldehyde in water. This involved the synthesis of a colorimetric sensor comprising gold nanoparticles with aminothiophenol (ATP-AuNPs). The prepared sensors are uniform in shape and size, with unique functional groups, can be simply and efficiently prepared, have good stability and can effectively detect formaldehyde. Furthermore, the -SH bond present in aminothiophenol can be linked with gold nanoparticles, and there are also amino groups present that can react with formaldehyde. Consequently, the ATP-AuNP sensor appears purple in the absence of formaldehyde, whereas upon the addition of formaldehyde, the color of the sensor changes to a light gray. The A_650_/A_530_ exhibits a high light absorption rate and it is relatively immune to external influences. This work presents a straightforward and effective approach for on-site and real-time detection of formaldehyde in water.

## 2. Materials and Methods

### 2.1. Experimental Reagents

Chloroauric acid (HAuCl_4_·3H_2_O), 4-aminothiophenol (4-ATP), 3-aminothiophenol (3-ATP), 2-aminothiophenol (2-ATP), sodium borohydride (NaBH_4_) and formaldehyde were purchased from Shanghai Titan Scientific Co., Ltd. (Shanghai, China), Ethanol, nitric acid and hydrochloric acid were sourced from local suppliers. All reagents were of analytical reagent grade and were used in their original state without undergoing any further purification. Throughout the course of this investigation, pure Millipore water was employed.

### 2.2. Synthesis of Aminobenzenethiol-Functionalized Gold Nanoparticles

All glassware was subjected to a cleaning process involving the use of a concentrated solution of hydrochloric acid and nitric acid (3:1, *v*/*v*), followed by a further rinse with distilled water.

The AuNPs were synthesized by the citrate reduction process [17]. In summary, 100 mL of 1% HAuCl_4_ was heated to boiling point. Subsequently, 2 mL of 1% trisodium citrate was added to the solution. The solution was heated until a color change to red was observed, after which it was removed from the oil bath pot and cooled to room temperature. The AuNPs were further functionalized (i.e., without centrifugation) in solutions containing 4-aminothiophenol (4-ATP-AuNPs, S1), 3-aminothiophenol (3-ATP-AuNPs, S2) and 2-aminothiophenol (2-ATP-AuNPs, S3), respectively. Initially, the pH of the AuNPs was adjusted to 3.0; this acidic AuNP solution was mixed with 10 mM aminothiophenol in a certain volumetric ratio (AuNPs/4-ATP = 9:1, AuNPs:3/ATP = 7:1 and AuNPs/2-ATP = 5:1) and then stirred for 30 min. The solution was finally filtered through a 0.45 µM filter paper and stored in a refrigerator at 4 °C. Following functionalization, the material retained its utility for quantitative analysis for at least 30 days, with no loss of performance.

### 2.3. Material Characterization

A small quantity of formaldehyde (100 μL, 0.01 M) was added to the synthesized ATP-AuNPs (900 μL). The colorimetric response of the formaldehyde concentration with three sensor elements was subjected to testing and recorded. Millipore water was used as a blank throughout the analysis. After 5 min of reaction, these solutions were characterized using the equipment listed below.

Transmission electron microscopy (TEM) and high-resolution TEM images of ATP-AuNPs and the formaldehyde addition were obtained using a JEOL JEM-2100F (JEO, Tokyo, Japan) at 160 kV. This enabled the diameters, morphology and aggregation of the particles to be measured. The synthesis of gold nanoparticles (ATP-AuNPs) and the subsequent alteration in particle size upon the addition of formaldehyde were quantified by dynamic light scattering (DLS) using the Zetasizer 3000HS (Malvern Instruments, Malvern, UK). Fourier transform infrared spectra (FT-IR) were obtained using the Nicolet 6700-NXR FTIR spectrometer (Thermo Fisher Scientific, Waltham, MA, USA) for the structural characterization of pure ATP, ATP-AuNPs and formaldehyde-added samples. The thermal stability of ATP-AuNPs was determined by thermogravimetric analysis using a thermogravimetric analyzer (TGA, TG-209, Thermo Fisher Scientific, Waltham, MA, USA) under nitrogen atmosphere. UV-Vis spectra were obtained using a Lambda 365 UV-Vis spectrometer (Perkin Elmer Instruments, Waltham, MA, USA) for the initial characterization of ATP-AuNPs and after the addition of formaldehyde [18].

## 3. Results and Discussion

### 3.1. Characterization of Aminobenzenethiol-Functionalized Gold Nanoparticles

**Figure 1:**
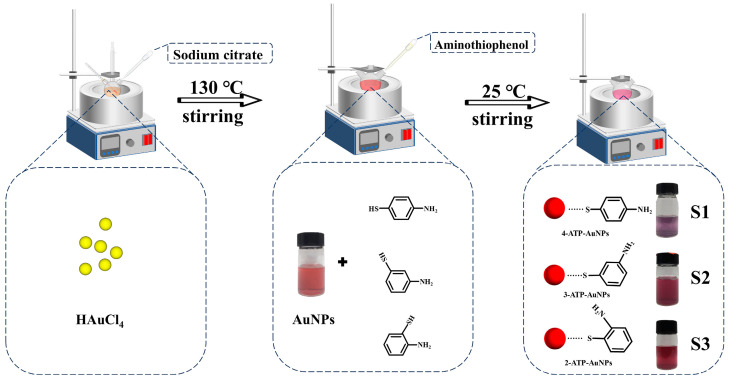
The processes of AuNP modification with the addition of S1, S2 and S3 solution.

Figure 1 depicts the synthesis of the aqueous suspension of AuNPs on three sensors (S1–S3) to detect formaldehyde. The synthesized AuNPs were capped with 4-ATP, 3-ATP and 2-ATP.

ATP-AuNPs were synthesized by mixing ATP solution with AuNP solution. ATP-AuNPs were synthesized due to the formation of a covalent bond between the -SH group of ATP and the AuNPs, resulting in the -NH_2_ group being released and reacting with the formaldehyde. The interactions of the AuNPs resulted in a change in the color of the solution, with the addition of ATP changing the AuNPs from ruby red to purple red, and the addition of formaldehyde changing the liquid from purple red to blue. Along with the color change, the maximum of the absorption spectrum shifted to a higher-wavelength region. This further indicates a change in the morphology of the nanoparticles [19].

#### 3.1.1. UV-Vis Spectroscopy Analysis

**Figure 2:**
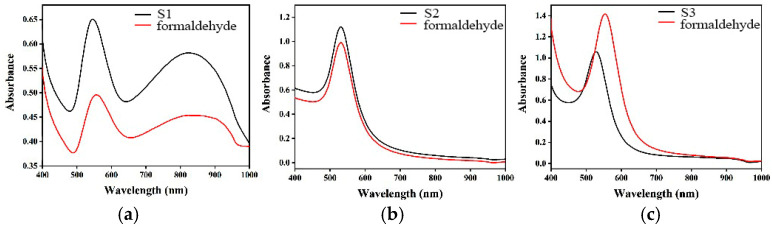
The changes in UV-Vis spectra of the S1 (**a**), S2 (**b**) and S3 (**c**) sensors upon the addition of formaldehyde.

Optical absorption spectra provide a clear representation of the dimensions and morphology of the AuNPs [20]. After 5 min of reaction, the absorption spectra were recorded using a Lambda 365 UV–visible spectrometer (Perkin Elmer Instruments, Waltham, MA, USA) in the wavelength range of 400 to 1000 nm. The addition of three different ligands resulted in a change in color from ruby red to violet red, which shifted the maximum of the absorption spectra of the AuNPs to a higher-wavelength region. Figure 2 depicts the maximum surface plasmon resonance (SPR) of the S1, S2 and S3 sensors at 530 nm, 535 nm and 527 nm, respectively. This indicates a change in the morphology of AuNPs [18].

#### 3.1.2. Dynamic Light Scattering and Transmission Electron Microscopy Analyses

**Figure 3:**
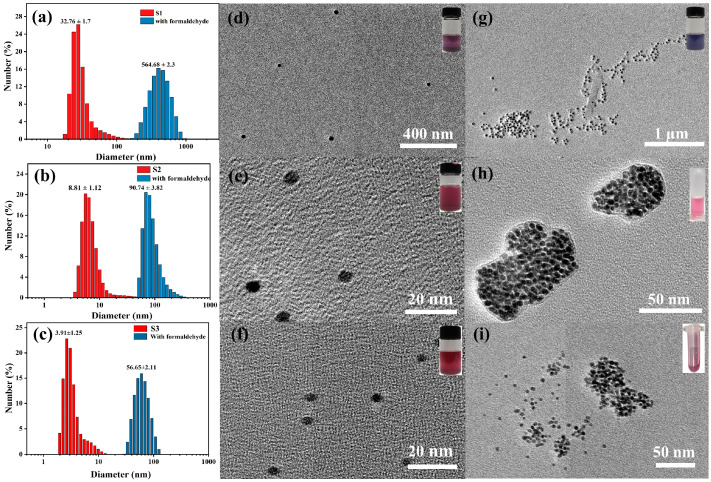
(**a**–**c**) Histograms of the diameters of the S1–S3 sensors (red) and formaldehyde-added (blue) nanoparticles. (**d**–**f**) TEM images of ATP-AuNP colorimetric S1–S3 sensors. (**g**–**i**) Aggregation states in the presence of 10 mM formaldehyde.

The surface morphology of the nanoparticles was determined by DLS and TEM as shown in Figure 3a–c. The particle sizes of the S1–S3 sensors were 32.76 ± 1.6 nm, 8.81 ± 1.12 nm and 3.91 ± 0.21 nm, respectively, and the shapes were basically spherical and uniform in size, as shown in Figure 3d–f. The TEM results in Figure 3g–i showed that the S1–S3 sensors were aggregated after the addition of formaldehyde [21,22]. At the same time, the color of the solution changed from red to purple red (Figure 3d–i). DLS analysis showed that the diameters of the formaldehyde-induced S1–S3 sensor aggregates increased by 17, 10 and 20 times, respectively.

**Figure 4:**
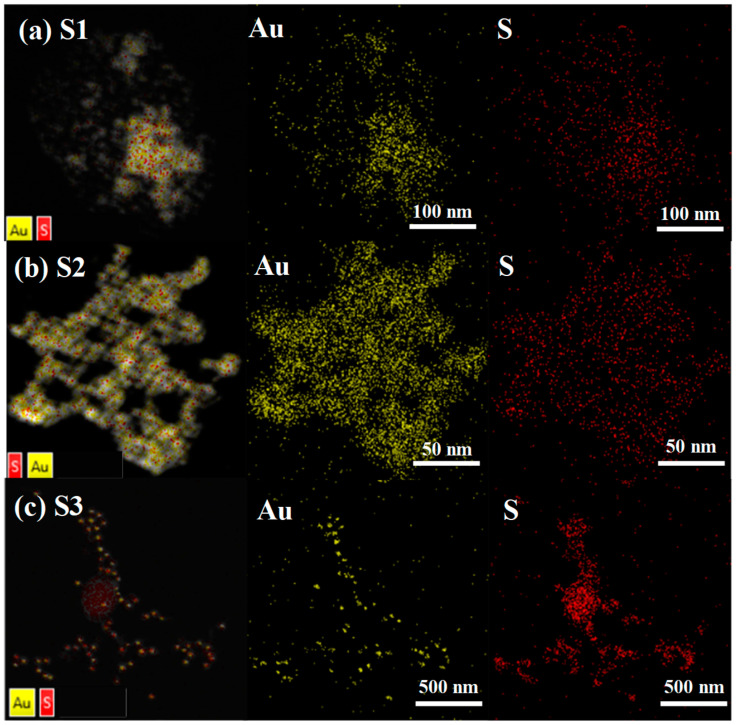
The EDS of ATP-AuNP colorimetric S1, S2 and S3 sensors in the presence of 110 mM formaldehyde is presented below. The Au and S symbols represent the EDS mapping of AuNPs and ATP-AuNPs mixed with formaldehyde, respectively.

The addition of formaldehyde reacts with the exposed amino groups to effectively cross-link the gold nanoparticle surface. This cross-linking forms a dense network of polymer chains on the surface of the gold nanoparticles. The gold nanoparticles are effectively encapsulated by the polymer network, preventing aggregation and stability of the colloidal solution. Sulfur atoms are likely to play a key role in anchoring this polymer network to the surface of gold nanoparticles (Figure 4).

#### 3.1.3. Fourier Transform Infrared Spectroscopy Analysis

**Figure 5:**
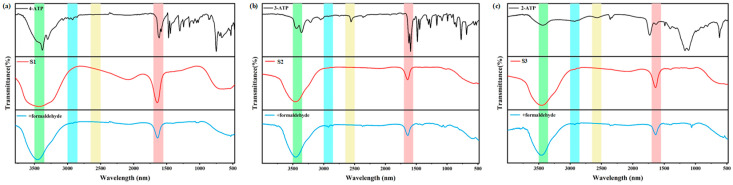
(**a**) FTIR spectra of 4-ATP, S1 and S1 with formaldehyde; (**b**) FTIR spectra of 3-ATP, S2 and S2 with formaldehyde; and (**c**) FTIR spectra of 2-ATP, S3 and S3 with formaldehyde.

In order to determine the nature of the functional groups on the surface of the prepared ATP-AuNPs, the surface structure of the ATP-AuNP samples in this study was characterized and analyzed in detail using infrared spectroscopy. According to Figure 5, it can be observed that the aminobenzenethiol gold nanoparticle system has a single property, and there is a stable bonding state between the Au-S bonds. FTIR spectroscopy [23] shows spectrally characteristic bands recorded in the spectral range of 500–3800 cm^−1^ for pure ATP (black curve), ATP-AuNPs (red curve) and ATP-AuNPs with formaldehyde (blue curve). The -SH stretching vibration of the free alkanethiol (-SH) is located at 2557 cm^−1^. However, no SH bonding of the alkanethiol is found in the curves of ATP-AuNPs, clearly indicating that the ATP molecule is disrupted upon binding to the gold nanoparticles and is bound to the gold via the -SH end [24]. This is also in good agreement with previous studies of alkanethiol modification of AuNPs by Murray and colleagues [25]. The peaks corresponding to N-H stretching and bending frequencies in the range of 3400–3500 cm^−1^ and 1550–1650 cm^−1^ remain in the presence of ATP and after ATP binding to AuNPs, suggesting that the presence of free NH_2_ could also be used for further reactions [26].

#### 3.1.4. pH Responsiveness

**Figure 6:**
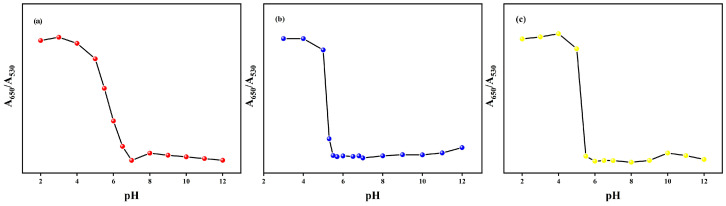
The ratios of UV absorption spectra (A_650_/A_530_) of the (**a**) S1, (**b**) S2 and (**c**) S3 sensors in the pH range of 2–12.

It has been reported that pH and environmental factors can significantly affect the aggregation behavior of plasma AuNPs in several scientific papers [27,28]. As shown in Figure 6, three different aminobenzenethiol gold nanoparticle colorimetric sensors exhibited similar pH response intervals. With the gradual decrease in ambient pH, these sensors showed an increasing trend at A_650_/A_530_. It is notable that the absorbance ratios of the S1, S2 and S3 sensors exhibit a significant change when the pH is reduced to 5. In alkaline environments, the concentration of hydroxide ions (OH^−^) in solution increases. As the nanogold surface is modified by amination with positive charges, electrostatic interactions occur between these positive charges and the increased OH^−^ ions.

This interaction results in the formation of a negatively charged shielding layer on the nanogold surface, which exerts an electrostatic stabilization effect [29]. This stabilizing effect is effective in preventing the aggregation of gold nanoparticles under alkaline conditions and maintaining their stability. Conversely, gold nanoparticles with ammonia roots become unstable and are prone to aggregation under acidic conditions.

#### 3.1.5. Time Stability Experiments of ATP-AuNP Colorimetric Sensor

**Figure 7:**
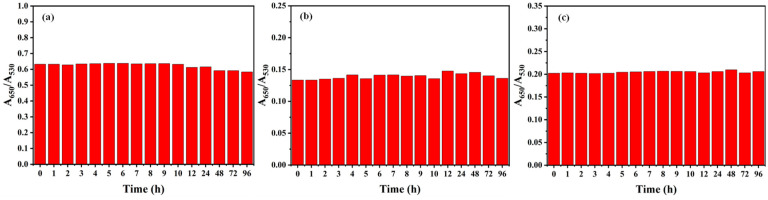
The ratios of UV absorption spectra (A_650_/A_530_) of (**a**) S1, (**b**) S2 and (**c**) S3 sensors for 96 h.

The time stability of the sensor was evaluated using a Lambda 365 UV-Vis spectrophotometer from PerkinElmer, Waltham, MA, USA. The solution was tested at different times to determine the time stability of the gold nanoparticle sensor over 96 h at room temperature. From Figure 7, it can be seen that the absorbance ratios (A_650_/A_530_) of the three sensors did not change significantly over the 96 h of test time, further demonstrating their stability. The results show that S3 is more stable than the other two sensors. In fact, the three sensors could be stored in a refrigerator at 4 °C for 90 days without any significant change. This demonstrates that the three sensors had removed excess unreacted feedstock during purification and that only stable aminophenol gold nanoparticles remained in the system.

#### 3.1.6. Response of the ATP-AuNP Colorimetric Sensor to Formaldehyde

**Figure 8:**
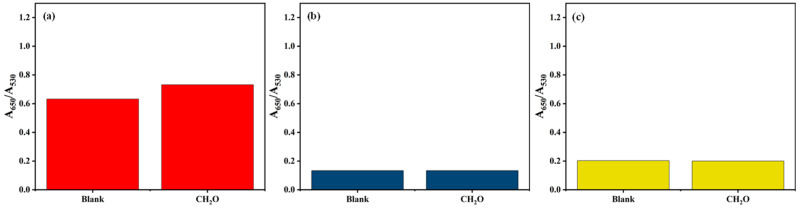
Plots of the responsiveness of the (**a**) S1, (**b**) S2 and (**c**) S3 sensors to formaldehyde.

In terms of the impact of formaldehyde on the induced aggregation of the S1, S2 and S3 sensors, it is evident from Figure 8 that the responsiveness of the S1 sensor to formaldehyde is significantly higher than the other two sensors. This superior responsiveness is advantageous in formaldehyde detection.

### 3.2. Interference Study

**Figure 9:**
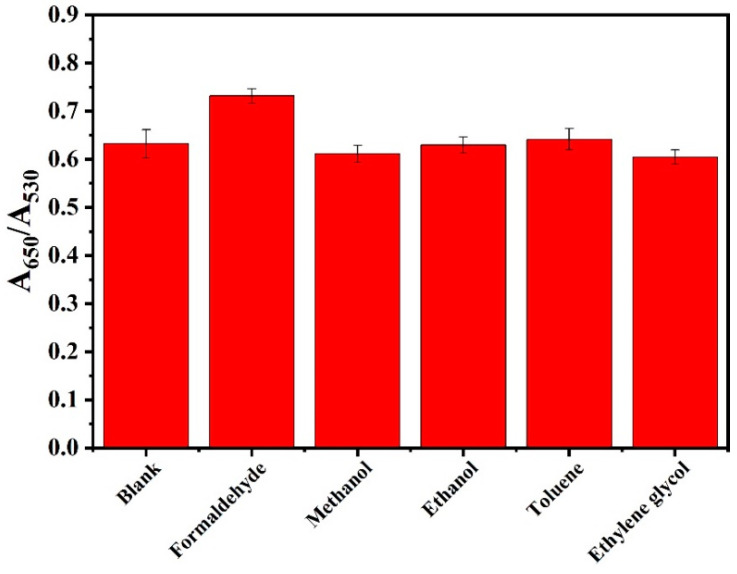
Plot of S1 sensor response to different solvents.

In order to ascertain whether foreign variables exert an influence on formaldehyde detection, a series of tests were conducted utilizing ATP-AuNPs spiked with formaldehyde, methanol, ethanol, toluene and ethylene glycol at a concentration of 10 mM. The results were expressed as A_650_/A_530_, as illustrated in Figure 9. It can be observed that the ratio remains largely unaltered when methanol and other samples are introduced, indicating that methanol and other samples do not exert a significant influence on the detection of formaldehyde at this concentration.

### 3.3. Formaldehyde Determination and Analysis of Real Water Samples

Formaldehyde effectively encapsulates gold nanoparticles by reacting with the amino group. This prevents aggregation and stabilizes the colloidal solution. The S1 sensor was used to detect the presence of formaldehyde. UV titration experiments were performed at room temperature and the characteristic spectral changes detected by the analytes were recorded with a UV–visible spectrometer. The color response of each sensor can be converted into an easily visualized spectrum. UV spectroscopy is the most commonly used method of signal output analysis in col-orimetric sensing strategies; It determines the difference between each analyte based on absorption wavelength shift or peak signal strength. Titration refers to a means of quantitative analysis, and to study the colorimetric response of ATP-AuNPs to formaldehyde, a titration curve of UV intensity was obtained to observe its concentration.

**Figure 10:**
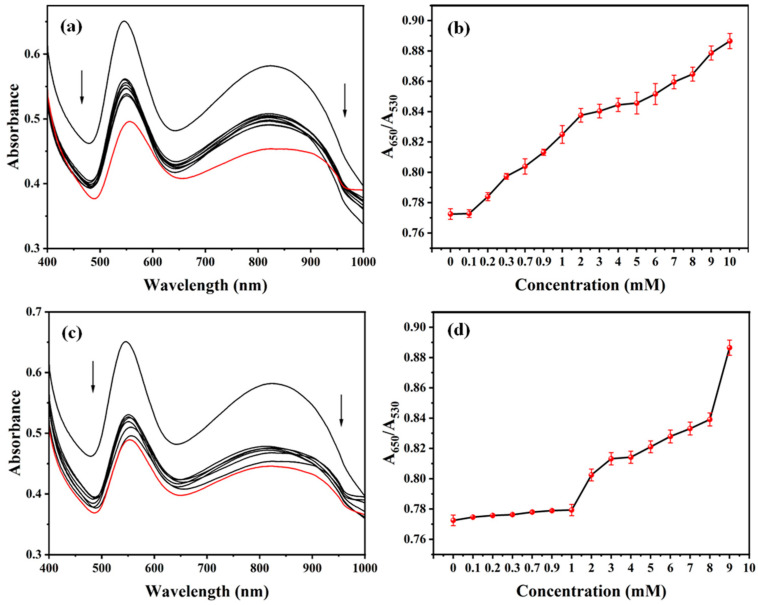
(**a**) The UV-Vis spectra and (**b**) changes in the extinction ratio (A_650_/A_530_) of the S1 sensor (ultrapure water) upon the addition of formaldehyde. (**c**) The UV-Vis spectra and (**d**) changes in the extinction ratio (A_650_/A_530_) of the S1 sensor (Li River water) upon the addition of formaldehyde. [formaldehyde] = 0–10 mM. Red indicates the absorbance when the formaldehyde concentration is 10 mM.

Subsequently, varying concentrations of formaldehyde (20 μL) were introduced to the S1 sensor (180 μL), and the UV-Vis absorption spectra were measured after 5 min of reaction to observe the change in UV spectra with the increase in formaldehyde concentration; the test was carried out in the same way using Lijiang River water to see whether the use of real water samples would have any effect on it. From Figure 10a,b, it can be seen that the detection of formaldehyde by the sensor under ultrapure water conditions increases gradually and slowly with increasing concentration, and the detection effect is positively correlated with the detection concentration. From Figure 10c,d, it can be seen that the sensor is still able to detect formaldehyde when using Li River water.

**Figure 11:**
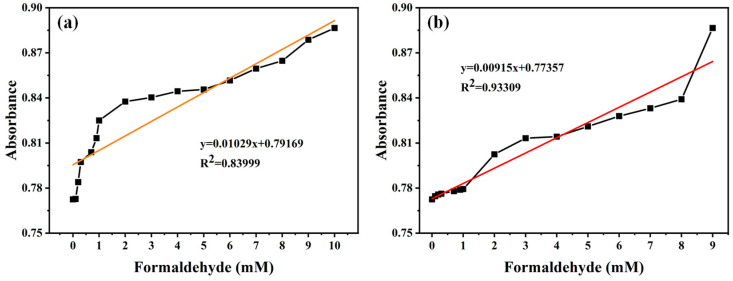
(**a**) Detection limit of the S1 sensor after addition of formaldehyde (ultrapure water) and (**b**) detection limit of the S1 sensor after addition of formaldehyde (river). [formaldehyde] = 0–10 mM.

Also, in order to verify the stability of the S1 sensor in a real-world setting, river water was selected as the diluent for formaldehyde, and the subsequent detection work was conducted accordingly. Therefore, the logical limit of detection (LOD) of the S1 sensor in ultrapure water for formaldehyde was 1.03 mM (Figure 11). This was calculated by using the calibration curve (y = 0.01029x + 0.079169, R^2^ = 0.83999) with data from Figure 10a through the following equation: LOD = 3σ/S (σ = 0.00352, s = 0.01029). The LOD in river water was calculated in the same way and was found to be 1.15 mM (y = 0.00915x + 0.77357, R^2^ = 0.93309, σ = 0.00352, s = 0.00915). The findings of this study indicate that the S1 sensor can be utilized in real-world settings, offering a novel approach for the detection of formaldehyde in aqueous solutions. The AuNP colorimetric assay demonstrated stability in real-world conditions, even in complex environments. The method exhibited high sensitivity and selectivity, enabling accurate detection of the target. Additionally, the assay’s rapid response characteristics make it well suited for on-site detection and environmental monitoring.

Table 1 compares the performance of the colorimetric sensor with that of other methods of formaldehyde detection reported in the literature. Despite the low detection limits of the formaldehyde detection methods mentioned, they are challenging to employ for real sample analysis due to the prolonged detection time, the necessity for analytical spectroscopic expertise and the intricate preparation of samples. In this study, gold nanomaterials were employed for the rapid detection of formaldehyde by the unaided eye. This approach offers several advantages over other detection techniques, including rapid detection and simple operation. However, it is limited by a lack of automation [30].

## 4. Conclusions

In conclusion, the self-assembly of aminophenol-modified gold nanoparticles with different relative positions of hydroxyl and amino groups has enabled the development of a fast-reacting and low-cost colorimetric sensor for the detection of formaldehyde. The S1 sensor was proven to be more effective in detecting formaldehyde compared to other sensors. A calibration curve was derived by configuring different concentrations of formaldehyde and the LOD was calculated to be 1.03 mM in ultrapure water and 1.15 mM in Li River water, which further demonstrated that the sensor enables straightforward yet rapid and precise analyses in both laboratory settings and real-world environments. This method is not only cost-effective but also avoids complex surface modification processes. In subsequent research, we intend to develop sensing techniques for more intricate environments, enhance the efficacy of formaldehyde identification and detection and conduct qualitative and quantitative analyses of an array of detectors. Furthermore, we plan to integrate artificial intelligence methodologies to facilitate more intelligent and streamlined detection processes.

## Figures and Tables

**Table 1:** Comparison of reported formaldehyde sensors with the current work.

Formaldehyde Sensors	LOD	Reference
Electrospinning Method	100 ppm	[31]
Hydrothermal Method	100 ppm	[32]
Solution Combustion Method	100 ppm	[33]
Fluorescence Sensor Method	100 ppm	[34]
Gold Nanocolorimetric Sensor	1.03 mM (30.9 ppm)	This work

## Data Availability

The original contributions presented in the study are included in the article, further inquiries can be directed to the corresponding author.
